# Serum creatinine to cystatin C ratio and cognitive function among middle-aged and older adults in China

**DOI:** 10.3389/fnagi.2022.919430

**Published:** 2022-09-09

**Authors:** Yueli Zhu, Zhongju Tan, Shumin Li, Feng Zhu, Chengfan Qin, Qin Zhang, Yunmei Yang

**Affiliations:** ^1^Department of Geriatrics, The First Affiliated Hospital, School of Medicine, Zhejiang University, Hangzhou, China; ^2^Key Laboratory of Diagnosis and Treatment of Aging and Physic-Chemical Injury Diseases of Zhejiang Province, The First Affiliated Hospital, School of Medicine, Zhejiang University, Hangzhou, China

**Keywords:** creatinine, cystatin C, cognitive function, sarcopenia, elders

## Abstract

**Background:**

The sarcopenia index (SI, serum creatinine/serum cystatin C × 100) is recently suggested to be a reliable marker for sarcopenia. It has been reported that sarcopenia is associated with poorer cognition. The purpose of this study was to evaluate the correlation between SI and cognitive function among middle-aged and older adults from the China Health and Retirement Longitudinal Study (CHARLS).

**Materials and methods:**

A total of 6,442 participants ≥45 years of age were enrolled in this study from CHARLS between 2011 and 2012. Cognitive function was assessed by interview-based measurements, including orientation and attention, episodic memory, visuo-construction, and the total cognitive function. SI was calculated by serum creatinine (mg/dL)/cystatin C (mg/L) × 100. One-way analysis of variance (ANOVA) was used to compare the differences among groups divided according to SI quartiles by gender. Both linear and logistic regression models were applied to investigate the relationship between SI and cognitive function.

**Results:**

After adjustment for potential confounders, we found SI was significantly and positively correlated with total cognitive function scores both in males and females [β = 0.014, 95% confidence interval (CI) 0.007 to 0.021, *P* < 0.001; β = 0.011, 95 CI% 0.003 to 0.018, *P* = 0.004; respectively]. Similarly, when the total cognitive function score was treated as a categorical variable according to quartiles in males and females, higher SI was related to better total cognitive function scores in both males and females [odds ratio (OR) = 1.147, 95% CI 1.028 to 1.279, *P* = 0.014; OR = 1.219, 95% CI 1.106 to 1.344, *P* < 0.001; respectively] following adjustment for confounders.

**Conclusions:**

Lower sarcopenia index was correlated with a higher prevalence of cognitive impairment among middle-aged and older adults in China.

## Introduction

With the global population continues to aged, the number of elderly individuals with mild cognitive impairment or dementia is dramatically rising in China and other countries (GBD 2016 Dementia Collaborators, 2019; Alzheimer’s Association, 2020; Jia et al., 2020). Mild cognitive impairment is considered to be the preclinical state of dementia and has gradually become an important global health problem, which affects people’s ability of learning and memory, reduces their quality of life, and has brought a heavy burden to their families and the whole country (Park et al., 2003; Liu et al., 2013). Thus, the early identification and prevention of cognitive impairment is of great significance in clinical practice.

Currently, more attention has been focused on searching blood biomarkers which is easy to obtain and cost-effective, since the positron emission tomography (PET) imaging is costly and lumbar puncture is unable to be generally accepted for diagnosing dementia (Feng et al., 2021). As we all know, serum creatinine and cystatin C are both biomarkers for glomerular filtration and renal function. Recently, a surrogate marker for sarcopenia called sarcopenia index (SI) has deen developed, which is defined as serum creatinine (mg/dL)/cystatin C (mg/L) × 100 and can be used to evaluate the skeletal muscle mass (Kashani et al., 2017; Barreto et al., 2019; Ren et al., 2022; Zheng et al., 2022). As we know, serum creatinine is the derivative of the skeletal muscle protein and the production of serum creatinine changes with skeletal muscle mass. In contrast, serum cystatin C is excreted by nucleated cells and the level is relatively constant when muscle mass varies. Thus, serum creatinine to cystatin C ratio can reflect the skeletal muscle mass (Kashani et al., 2017). Lower serum creatinine and cystatin C ratio is related to lower skeletal muscle mass and the higher likelihood of sarcopenia. Prior studies have reported that sarcopenia was associated with cognitive decline in elderly adults (Cabett Cipolli et al., 2019; Peng et al., 2020). However, up to now, no studies have been conducted to assess whether SI, a novel biomarker of sarcopenia, is related to cognitive function.

The aim of our study was to investigate the gender-specific correlations between SI and cognitive function using data from the China Health and Retirement Longitudinal Study (CHARLS), which was a national representative survey.

## Materials and methods

### Study population

This retrospective cross-sectional study was conducted using the CHARLS database. CHARLS is a nationally representative longitudinal study of the population aged 45 and older and their spouses, and covers a broad range of areas, including demographic information, family structure, health status and functioning, healthcare and insurance, work, retirement and pension, income, and so on (Zhao et al., 2013, 2014). The baseline survey of CHARLS was conducted between June 2011 and March 2012 and the participants were followed every 2 years using a computer-assisted personal interview. The study design and protocol were approved by the Biomedical Ethics Review Committee of Peking University (IRB00001052-11015), and informed consent was obtained from all participants. More information can be found in the CHARLS project website.^1^ In this study, the baseline survey data were collected and used. Participants were excluded if they: (1) aged <45 years; (2) without complete data, such as serum creatinine, cystatin C, and cognitive function scores; and (3) had the history of kidney disease or an estimated glomerular filtration rate (eGFR) of less than 30 mL/(min × 1.73 m^2^) by using the Chinese-based equation (Li et al., 2019).

### Cognitive assessment

Cognitive function was assessed from the following three dimensions: (1) Orientation and attention. Telephone Interview of Cognitive Status (TICS) was used to measure the participants’ mental status, including an awareness of the serial subtraction of 7 from 100 (up to five times), date (year, month, day), day of the week, and season of the year (Huang and Zhou, 2013). The orientation and attention score ranged from 0 to 10. (2) Episodic memory. Interviewers read a list of 10 random words, and participants were asked to immediately recall as many words as possible (immediate recall). Participants were asked to recall the same words as many as possible about 5 mins later (delayed recall) (Luo et al., 2021). The episodic memory score was calculated as the average number of immediate and delayed word recalls and ranged from 0 to 10. (3) Visuo-construction. The figure drawing test was used to assess the participates’ visuospatial abilities. Interviewers showed a picture of two overlapped pentagons and asked participates to draw a similar figure on paper. They were able to receive a score of 1 after successfully completing the task, and otherwise a score of 0 if they failed. The visuo-construction score ranged from 0 to 1 (Huang and Zhou, 2013). The total cognitive function score was calculated by the summation of these three dimensions, ranging from 0 to 21.

### Serum creatinine and cystatin C

According to a standard protocol, venous blood samples of each participant were collected after an overnight fast by trained staff from the Chinese Center for Disease Control and Prevention (Chinese CDC). After collection, the blood samples were transported to the local laboratories at 4°C. After the blood samples were separated into plasma and buffy coat, they were stored in separate cryovials. And then these cryovials were immediately stored frozen at −20°C and transported to the Chinese CDC in Beijing within 2 weeks. Finally, they were stored at −80°C until assay at Capital Medical University laboratory. Serum creatinine was measured using a rate-blanked and compensated Jaffe creatinine method while the particle-enhanced turbidimetric assay method was used for cystatin C.

### Other variables

Information including age, gender, height, weight, hand grip strength, educational level, marital status, residence (urban or rural), smoking and alcohol drinking status, and chronic diseases (hypertension, diabetes, dyslipidemia, cancer, stroke, heart problems, lung disease, liver disease, and digestive disease) was gathered. Hand grip strength was evaluated using the dynamometer (Yuejian WL-1000). Each hand was measured twice and grip strength was set as the maximal value among the four measurements from both hands. These diseases were self-reported physician-diagnosed. Body mass index (BMI) was defined as weight in kilograms divided by height in meters squared.

### Statistical analysis

The continuous variables were presented with means and standard deviation (SD), while numbers and percentages were used for categorical variables. For continuous variables, between-group comparisons were performed using the independent sample *t*-test and one-way analysis of variance (ANOVA) was used to compare the differences among four groups. And the chi-squared test was performed to detect the differences among groups for categorical variables. Based on the quartile boundaries of SI, males were grouped as “< 71.91,” “≥ 71.91 and < 82.75,” “≥ 82.75 and < 94.74,” and “≥ 94.74,” and females were grouped as “< 62.53,” “≥ 62.53 and < 72.02,” “≥ 72.02 and < 81.69,” and “≥ 81.69.”

Unadjusted and adjusted linear regression models were applied to explore the correlation between SI and cognitive function, with SI as the independent variables and cognitive function scores as the dependent variables. Unadjusted and adjusted logistic regression models were further performed to examine the relationship, while SI quartiles were treated as the independent variables and cognitive function (Q3 vs. Q1) was treated as the dependent variables. These models were adjusted for potential confounders, including age, BMI, educational level, smoking, drinking, and the status of each chronic disease (covering hypertension, diabetes, dyslipidemia, cancer, stroke history, heart problems, lung disease, liver disease, and digestive disease). Odds ratio (OR) and 95% confidence interval (CI) were calculated by logistic regression analysis.

All statistical analyses were performed using IBM SPSS Statistics 26. A *P*-value of less than 0.05 was considered statistically significant.

## Results

A total of 6,442 participants were included in this study and 3007 (46.7%) of them were males. The mean age was 61.5 years old (SD = 9.7) and 60.0 years old (SD = 10.0) in males and females, respectively. There were significant differences in age, BMI, education level, marital status, rural residence, smoking and alcohol drinking status, chronic diseases, hand grip strength, SI, and cognitive function scores between males and females. These characteristics were presented in Table 1.

**TABLE 1:** Demographic data of the study participants.

Characteristics	Male (*N* = 3007)	Female (*N* = 3435)	*P*
Age (years), mean ± SD	61.5 ± 9.7	60.0 ± 10.0	<0.001
BMI (kg/m^2^), mean ± SD	22.9 ± 3.7	23.9 ± 4.1	<0.001
Education, *N* (%)			<0.001
No formal education	1062 (35.3)	2119 (61.7)	
Primary school	825 (27.4)	612 (17.8)	
Middle or high school	1061 (35.3)	667 (19.4)	
College or above	59 (2.0)	37 (1.1)	
Married (*N*, %)	2695 (89.6)	2839 (82.6)	<0.001
Living in rural area (*N*, %)	1993 (66.3)	2183 (63.6)	0.022
Smoking (*N*, %)	2243 (74.6)	277 (8.1)	<0.001
Alcohol drinking status (*N*, %)			<0.001
Never	1355 (45.1)	3023 (88.0)	
Less than once a month	305 (10.1)	164 (4.8)	
More than once a month	1347 (44.8)	248 (7.2)	
Hypertension (*N*, %)	696 (23.1)	914 (26.6)	0.001
Diabetes (*N*, %)	141 (4.7)	222 (6.5)	0.002
Dyslipidemia (*N*, %)	236 (7.8)	326 (9.5)	0.020
Cancer (*N*, %)	21 (0.7)	30 (0.9)	0.429
Stroke history (*N*, %)	76 (2.5)	60 (1.7)	0.030
heart problems (*N*, %)	297 (9.9)	453 (13.2)	<0.001
Lung disease (*N*, %)	378 (12.6)	295 (8.6)	<0.001
Liver disease (*N*, %)	111 (3.7)	105 (3.1)	0.158
Digestive disease (*N*, %)	628 (20.9)	816 (23.8)	0.006
Hand grip strength (Kg)	38.5 ± 9.5	26.5 ± 7.3	<0.001
**Laboratory data, mean ± SD**			
Serum creatinine (mg/dL)	0.88 ± 0.18	0.70 ± 0.15	<0.001
Serum cystatin C (mg/L)	1.07 ± 0.25	0.97 ± 0.25	<0.001
SI	84.5 ± 20.0	74.2 ± 18.7	<0.001
**Cognitive function, mean ± SD**			
Orientation and attention score	7.0 ± 2.7	5.6 ± 3.0	<0.001
Episodic memory score	3.2 ± 1.9	3.0 ± 2.0	<0.001
Visuo-construction score	0.7 ± 0.5	0.5 ± 0.5	<0.001
Total cognitive function score	10.9 ± 4.1	9.1 ± 4.6	<0.001

SD, standard deviation; BMI, body mass index; SI, sarcopenia index.

**FIGURE 1:**
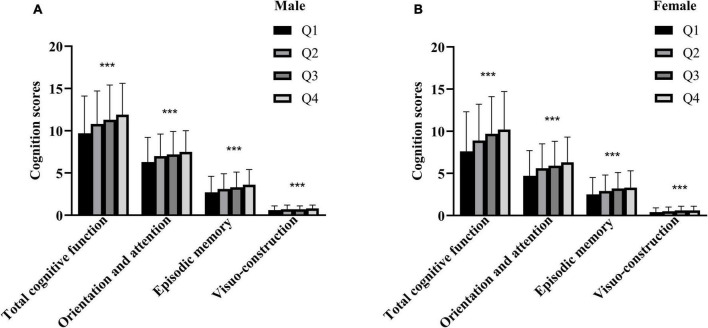
Average cognition scores between groups divided by SI quartiles. **(A)** In the males, **(B)** in the females. ***The cognition scores among the four groups were significantly different (*P* < 0.001).

Then, SI was treated as a categorical variable based on the quartile cut-off points in males and females, respectively (Table 2). In both males and females, we found that the mean age was lower and the mean BMI was higher in the Q4 group compared with the other groups (all *P* < 0.001). What’s more, the proportions of living in rural area and the low level of education were lower in the Q4 group (all *P* < 0.001). And the percentages of smoking were lower in the Q4 group of males (*P* = 0.005). Of note, the proportion of drinking was higher in the Q4 group of males (*P* < 0.001), while the similar phenomenon was not observed in females (*P* = 0.893). In particular, the hand grip strength was the greatest in the Q4 group compared with the other groups (all *P* < 0.001), regardless of gender. Thus, the positive relationship between SI and sarcopenia in this study was confirmed, which was consistent with the previous literatures. And the mean total cognitive function score of the Q4 group was the highest among the four groups in males, as well as in females (all *P* < 0.001, Figure 1).

**TABLE 2:** Characteristics of participants according to SI quartiles.

Characteristics	Quartiles of SI Male (*N* = 3007)					Quartiles of SI Female (*N* = 3435)				
		
	Q1*	Q2*	Q3*	Q4*	*P*	Q1^#^	Q2^#^	Q3^#^	Q4^#^	*P*
Age (years)	65.8 ± 10.2	62.7 ± 9.3	60.0 ± 8.8	57.4 ± 8.3	<0.001	64.9 ± 10.5	61.0 ± 9.6	57.7 ± 8.8	56.7 ± 9.0	<0.001
BMI (kg/m^2^)	21.7 ± 3.7	22.6 ± 3.5	23.2 ± 3.3	24.0 ± 3.7	<0.001	23.4 ± 4.5	23.6 ± 3.9	24.2 ± 3.9	24.4 ± 4.0	<0.001
Primary school or below, *N* (%)	539 (71.3)	479 (64.0)	453 (60.1)	416 (55.5)	<0.001	732 (87.4)	744 (85.0)	648 (76.5)	607 (69.4)	<0.001
Living in rural area (*N*, %)	571 (75.5)	524 (70.1)	471 (62.5)	427 (57.0)	<0.001	549 (65.5)	574 (65.6)	560 (66.1)	500 (57.1)	<0.001
Smoking (*N*, %)	593 (78.4)	565 (75.5)	556 (73.7)	529 (70.6)	0.005	80 (9.5)	77 (8.8)	65 (7.7)	55 (6.3)	0.071
Alcohol drinking (*N*, %)	380 (50.3)	387 (51.7)	439 (58.2)	446 (59.5)	<0.001	101 (12.1)	99 (11.3)	106 (12.5)	106 (12.1)	0.893
Chronic disease^†^ (*N*, %)	395 (52.2)	415 (55.5)	405 (53.7)	402 (53.7)	0.662	479 (57.2)	532 (60.8)	453 (53.5)	490 (56.0)	0.021
Hand grip strength (Kg)	34.5 ± 9.5	37.6 ± 9.0	39.8 ± 9.2	42.0 ± 8.5	<0.001	24.0 ± 7.2	26.4 ± 7.0	27.5 ± 7.1	28.0 ± 7.3	<0.001
Orientation and attention score	6.3 ± 2.9	7.0 ± 2.6	7.2 ± 2.7	7.5 ± 2.5	<0.001	4.7 ± 3.0	5.6 ± 2.9	5.9 ± 2.9	6.3 ± 3.0	<0.001
Episodic memory score	2.7 ± 1.9	3.1 ± 1.8	3.3 ± 1.8	3.6 ± 1.8	<0.001	2.5 ± 2.0	2.9 ± 1.9	3.2 ± 1.9	3.3 ± 2.0	<0.001
Visuo-construction score	0.6 ± 0.5	0.7 ± 0.5	0.7 ± 0.4	0.8 ± 0.4	<0.001	0.4 ± 0.5	0.5 ± 0.5	0.6 ± 0.5	0.6 ± 0.5	<0.001
Total cognitive function score	9.7 ± 4.4	10.8 ± 3.9	11.3 ± 4.1	11.9 ± 3.7	<0.001	7.6 ± 4.7	8.9 ± 4.3	9.7 ± 4.4	10.2 ± 4.5	<0.001

Values are presented as mean ± standard deviation (SD) if not otherwise specified. SI, sarcopenia index; BMI, body mass index. In male: Q1* < 71.91, 71.91 ≤ Q2* < 82.75, 82.75 ≤ Q3* < 94.74, Q4* ≥ 94.74. In female: Q1^#^ < 62.53, 62.53 ≤ Q2^#^ < 72.02, 72.02 ≤ Q3^#^ < 81.69, Q4^#^ ≥ 81.69. Chronic disease^†^: including hypertension, diabetes, dyslipidemia, cancer, stroke, heart problems, lung disease, liver disease, and digestive disease.

To further investigate the relationship between SI and cognition, linear regression models were performed (Table 3). In the unadjusted model, higher SI was correlated with better cognitive function in males and females (all *P* < 0.001), including the three dimensions of orientation and attention, episodic memory, and visuo-construction. After adjustment for confounders, SI was significantly and positively related to better orientation and attention, episodic memory, and total cognitive function scores in males (β = 0.007, 95 CI% 0.002 to 0.012, *P* = 0.005; β = 0.007, 95 CI% 0.003 to 0.010, *P* < 0.001; β = 0.014, 95 CI% 0.007 to 0.021, *P* < 0.001; respectively), while higher SI was only significantly correlated with higher orientation and attention, and total cognitive function scores in females (β = 0.008, 95 CI% 0.003 to 0.013, *P* = 0.003; β = 0.011, 95 CI% 0.003 to 0.018, *P* = 0.004; respectively).

**TABLE 3:** Linear regression models for correlations between SI and cognitive function scores.

Outcome	Model 1		Model 2	
		
	β (95% CI)	*P*	β (95% CI)	*P*
**Male**				
Orientation and attention score	0.020 (0.015–0.025)	<0.001	0.007 (0.002–0.012)	0.005
Episodic memory score	0.016 (0.013–0.019)	<0.001	0.007 (0.003–0.010)	<0.001
Visuo-construction score	0.003 (0.002–0.003)	<0.001	0.001 (0.000–0.001)	0.149
Total cognitive function score	0.038 (0.031–0.045)	<0.001	0.014 (0.007–0.021)	<0.001
**Female**				
Orientation and attention score	0.025 (0.019–0.030)	<0.001	0.008 (0.003–0.013)	0.003
Episodic memory score	0.013 (0.010–0.017)	<0.001	0.002 (−0.001 to 0.006)	0.149
Visuo-construction score	0.003 (0.002–0.004)	<0.001	0.001 (0.000–0.001)	0.239
Total cognitive function score	0.041 (0.033–0.049)	<0.001	0.011 (0.003–0.018)	0.004

SI, sarcopenia index; CI, confidence interval. Model 1: unadjusted model. Model 2: adjusted model including age, BMI, educational level, smoking, drinking and chronic diseases (hypertension, diabetes, dyslipidemia, cancer, stroke history, heart problems, lung disease, liver disease, and digestive disease).

When the total cognitive function score was treated as a categorical variable according to quartiles in males and females, respectively (Q3 vs. Q1), the positive correlation between SI and cognitive function scores was observed in unadjusted logistic regression models (OR = 1.389, 95% CI 1.264 to 1.526, *P* < 0.001; OR = 1.480, 95% CI 1.358 to 1.614, *P* < 0.001; respectively; Table 4). Following adjustment for confounders, the relationship remained significant in both males and females (OR = 1.147, 95% CI 1.028 to 1.279, *P* = 0.014; OR = 1.219, 95% CI 1.106 to 1.344, *P* < 0.001; respectively; Table 4).

**TABLE 4:** Binary logistic regression models for correlations between SI and cognitive function.

Outcome	Model 1		Model 2	
		
	OR (95% CI)	*P*	OR (95% CI)	*P*
**Male**				
Total cognitive function score (Q3 vs. Q1)*	1.389 (1.264–1.526)	<0.001	1.147 (1.028–1.279)	0.014
**Female**				
Total cognitive function score (Q3 vs. Q1)^#^	1.480 (1.358–1.614)	<0.001	1.219 (1.106–1.344)	<0.001

SI, sarcopenia index; OR, odds ratio; CI, confidence interval. *Based on the quartiles of total cognitive function score in males. 11.5 ≤ Q3 < 14; Q1 < 8.5. ^#^Based on the quartiles of total cognitive function score in females. 9 ≤ Q3 < 12.5; Q1 < 6. Model 1: unadjusted model. Model 2: adjusted for confounders including age, BMI, education level, smoking, drinking and chronic diseases (hypertension, diabetes, dyslipidemia, cancer, stroke history, heart problems, lung disease, liver disease, and digestive disease).

## Discussion

In this study, we found that higher SI was independently correlated with better cognitive function in both linear and logistic regression models after adjusted for confounding factors, irrespective of gender. To our knowledge, it is the first study to evaluate the relationship between SI and cognitive function.

Sarcopenia is characterized by low skeletal muscle mass and low muscle function (including muscle strength or physical performance) which occurs during normal aging (Cruz-Jentoft et al., 2010; Wiedmer et al., 2021). And recently, the diagnosis of sarcopenia is mainly based on the presence of low muscle strength and low muscle quantity or quality (Cruz-Jentoft et al., 2019). It has been reported to increase the risks of frailty, falling, cognitive impairment, functional decline, and even mortality (Cruz-Jentoft et al., 2019). A growing number of studies have proposed that SI is a credible biomarker for skeletal muscle mass (Kashani et al., 2017; Ichikawa et al., 2020; Lin et al., 2020). And this prompted us to investigate the relationship between SI and cognitive function, since SI was easy to obtain in clinic and can be widely applied.

Existing evidence has revealed that there is a clear gender difference in SI (Lin et al., 2020; Okubo et al., 2022; Tang et al., 2022). And it can be understood as the differences of serum creatinine concentration across genders since the skeletal muscle mass of males is usually greater than of females. Similarly, the diagnostic criteria for sarcopenia in males and females are also not same. Hence, we conducted the study on males and females separately.

Based on the linear and logistic regression models, evidence from our study showed that lower SI was significantly correlated with cognitive decline in males and females. To interpret this relationship, we mainly attribute it to the bidirectional effects of sarcopenia and cognition since SI is regarded as a promising biomarker for sarcopenia. The underlying mechanisms for the relationship between sarcopenia and cognitive impairment have not been fully understood. First, elevated levels of plasma pro-inflammatory cytokines are found in dementia patients, such as interleukin-6 (Licastro et al., 2000). And this could negatively influence muscle protein metabolism through direct catabolic or indirect mechanisms (Dalle et al., 2017). Besides, elderly adults with cognitive impairment are prone to stay at home and reduce food intake, which may lead to poor musculoskeletal health, like loss of muscle mass and muscle strength (Beaudart et al., 2019). Finally, reduced physical exercise, generated by sarcopenia, may decrease the level of the brain-derived neurotrophic factor which is related to learning and memory (Kang and Schuman, 1995; Erickson et al., 2011). The exact mechanisms of the relationship between SI and cognitive function remains to be elucidated by future biological studies.

A major strength of this study was a large number of participants from a nationally representative database, providing more solid conclusions. Meanwhile, this study was clinically innovative and possessed values for clinical applications. And we adjusted as much confounders as possible in our study to improve the reliability of results. However, some potential limitations in this study should to be considered. First, due to the restrictions of CHARLS database, neurocognitive functioning was not assessed by the Mini-Mental State Examination (MMSE) and the Montreal Cognitive Assessment (MoCA), which are the two most frequently used screening tools in cognition. Future studies with more formal neuropsychological assessments are required to further confirm the relationship between SI and cognitive function. Second, the exclusion of participants with incomplete data may introduce some selection bias. But ultimately, the number of included participants is large enough which can mitigate the effect of selection bias. Third, the diagnoses of participants’ chronic diseases were defined based on self-reported. And some participants may be unaware of their diseases. Confounders associated with sarcopenia, including nutrition and exercise, were not adjusted in this study since the CHARLS database didn’t provide the nutrition information and had a large proportion of missing data about exercise information. Finally, this study mainly focused on participants aged 45 years and older in China. Thus, it remained unclear whether the conclusions could be fully generalized to the populations in other countries.

## Conclusion

In summary, we assessed the correlation of SI and cognitive function in a middle-aged and older Chinese population from the CHARLS database. And it showed that higher SI was significantly correlated with better cognitive function in both males and females. Further studies should be conducted to validate the correlation between SI and cognitive function and explore the exact mechanisms.

## Data availability statement

Publicly available datasets were analyzed in this study. This data can be found here: http://charls.pku.edu.cn/.

## Ethics statement

Ethical approval was not provided for this study on human participants because the data was from the China Health and Retirement Longitudinal Study (CHARLS), which was approved by the Biomedical Ethics Review Committee of Peking University (IRB00001052-11015). The patients/participants provided their written informed consent to participate in this study.

## Author contributions

YZ and YY: study concept and design. YZ, ZT, SL, FZ, and CQ: acquisition, analysis, and interpretation of data. YZ: drafting the manuscript. YY and QZ: critical revision. All authors approved the final manuscript.

## Footnotes

1
http://charls.pku.edu.cn/

## Abbreviations

ANOVA: analysis of variance
BMI: body mass index
CDC: center for disease control and prevention
CHARLS: China health and retirement longitudinal study
CI: confidence interval
MMSE: mini-mental state examination
MoCA: Montreal cognitive assessment
OR: odds ratio
PET: positron emission tomography
SD: standard deviation
SI: sarcopenia index
TICS: telephone interview of cognitive status.
